# Physical Mechanism of Nanocrystalline Composite Deformation Responsible for Fracture Plastic Nature at Cryogenic Temperatures

**DOI:** 10.3390/nano14080723

**Published:** 2024-04-20

**Authors:** Jianyong Qiao, Ivan Vladimirovich Ushakov, Ivan Sergeevich Safronov, Ayur Dasheevich Oshorov, Zhiqiang Wang, Olga Vitalievna Andrukhova, Olga Vladimirovna Rychkova

**Affiliations:** 1 School of Energy and Mining Engineering, China University of Mining and Technology (Beijing), Beijing 100083, China; qjy@bupt.edu.cn; 2 Physics Department, National University of Science and Technology “MISIS”, Moscow 119049, Russia; issafronov@yandex.ru (I.S.S.);; 3 China-Russia Dynamics Research Center, China University of Mining and Technology (Beijing), Beijing 100083, China; wzhiqianglhm@126.com

**Keywords:** nanomaterials, condensed matter physics, nanotechnology, cryogenic temperatures, physical deformation mechanism

## Abstract

In this work, we consider the physical basis of deformation and fracture in layered composite nanocrystalline/amorphous material–low-melting crystalline alloy in a wide temperature range. Deformation and fracture at the crack tip on the boundary of such materials as nanocrystalline alloy of the trademark 5BDSR, amorphous alloy of the trademark 82K3XSR and low-melting crystalline alloy were experimentally investigated. The crack was initiated by uniaxial stretching in a temperature range of 77–293 K. A theoretical description of the processes of deformation and fracture at the crack tip is proposed, with the assumption that these processes lead to local heating and ensure the plastic character of crack growth at liquid nitrogen temperatures. The obtained results improve the theoretical understanding of the physics of fracture at the boundary of nanocrystalline and crystalline alloys in a wide temperature range. The possibility of preserving the plastic nature of fracture in a thin boundary layer of crystalline–nanocrystalline material at cryogenic temperatures has been experimentally shown.

## 1. Introduction

Amorphous and nanocrystalline metal alloys have unique physical and mechanical properties [1,2,3]. The study of such materials is of considerable interest for modern condensed matter physics [4,5,6]. The improvement of the physical and mechanical properties of amorphous/nanocrystalline materials is possible with the creation of layered composites. The development of effective methods for creating metal–metalloid composites with specified characteristics requires an understanding of the peculiarities and kinetics of the deformation/destruction of composite materials, as well as investigating the formation regularities of their mechanical properties over a wide temperature range [7,8,9,10].

The study of the physical mechanism describing the deformation process’s initiation in the contact zone of nanocrystalline/amorphous and crystalline metal alloys at ultra-low temperatures is an urgent task in condensed matter physics, as it is important for understanding the physics of strength, plasticity and destruction of heterogeneous nanostructures under conditions of cryogenic embrittlement [11,12].

The effects of embrittlement in crystalline materials are well studied; they depend on the type of crystal lattice and the chemical composition of the samples [10,13,14,15]. In the case of amorphous metal alloys, the deformation pattern changes from homogeneous to heterogeneous as the temperature decreases. Microhardness and yield strength for amorphous metal alloys can be divided into thermal and non-thermal components, the non-thermal component depending on the chemical composition and crystal lattice, while the temperature dependence of microhardness is not affected practically by the structural and chemical composition [16].

Currently, there is a need for composite materials with high strength characteristics [17,18,19]. In some cases, additional requirements are imposed on the composites, including the ability to maintain performance properties at low temperatures (for example, for Arctic/Antarctic regions, deep space, etc.) [20,21,22].

The creation and research of the mechanical properties exhibited by layered composites based on nanocrystalline/amorphous metal films and low-melting-point metal fusible alloys is promising [23]. On the one hand, the choice of low-melting alloys allows for preserving the nanocrystalline/amorphous structure of films during the fusion of materials. On the other hand, a thin layer with unusual physical and mechanical properties is formed in the contact zone due to the reciprocal influence of nanocrystalline/amorphous and crystalline structures [24,25,26,27]. To analyze the features of the deformation and fracture process in such composites, it is necessary to conduct experimental studies in the widest possible temperature range. However, even relatively low temperatures (>473 K) complicate the conduction of research due to structural relaxation and/or crystallization in a nanocrystalline/amorphous metal alloy [28,29]. Therefore, the experimental study of deformation and fracture under uniaxial tension of composite samples at room and cryogenic temperatures becomes important [30]. In addition, the combination of various types of components in hybrid metal composite materials is known to provide an increase in physicochemical characteristics [9,10,17,18,19,20]. This approach is the most promising for the involved class of nanostructured materials.

The main purpose of this study is to create a composite compound that retains strength at cryogenic temperatures, as well as to research the physical mechanism of deformation and destruction in nano-areas at the boundary between crystalline and nanocrystalline/amorphous metal alloys at cryogenic embrittlement.

## 2. Materials and Methods

The physical properties of layered metal composites are expected to be considerably determined by the characteristics of the contact zone formed during fusion of a nanocrystalline/amorphous ribbon and a crystalline alloy. Obviously, a significant influence of these contact zone properties on the mechanical characteristics of the sample as a whole will be observed at sufficiently small thicknesses of the composite layers. The preparation of samples matching this condition will make it possible to identify the features of the deformation and destruction processes of such materials.

Products from the Ashinsky Metallurgical Plant were used for fusion: nanocrystalline tape of the 5BDSR brand (the main parameters of the alloy are provided on the website https://amet.ru, accessed on 18 July 2023): 77% Fe + 1% Cu + 3% Nb + 13% Si + 3% B (wt. %) with an initial crystallization temperature of 753 K and amorphous tape 82K3XSR (the main parameters of the alloy are provided on the website https://amet.ru/23/, accessed on 18 July 2023): 83.7% Co + 3.7% Fe + 3.2% Cr + 9.4% Si (wt. %) with an initial crystallization temperature of 733 K. It has been experimentally established that embrittlement of amorphous tapes begins when the tapes are heated to temperatures greater than 550 K. Samples of tapes (5BDSR and 82K3XSR) with a width of 20–25 mm and a length of 100 mm were prepared. The average thickness of the tape, according to the technical documentation, was 30 microns.

For a low-melting crystalline binder, the following items were used: solder POSK 50-18 (https://zubr.ru/, accessed on 25 September 2023) 50% Sn + 32% Pb + 18% Cd (wt. %) (melting point 456 K); Wood alloy (https://www.sigmaaldrich.com/RU/en, accessed on 25 September 2023): 50% Bi + 25% Pb + 12.5% Sn + 12.5% Cd (melting point 341 K) and alloy Sn63Pb37 in the form of an indelible solder paste SD-318 (the main parameters of the alloy are provided on the website https://www.chipdip.ru/, accessed on 25 September 2023) (melting point 456 K).

The selected low-melting materials have specific temperature dependencies of microhardness and brittleness as well as low melting points. This allows us to minimize structural relaxations and prevent crystallization of the amorphous phase (recrystallization, structural relaxation) during the process of fusing with a nanocrystalline/amorphous tape.

It has been experimentally found that solder POSK 50-18 and Wood alloy wet the amorphous alloy grade 82K3XSR (PJSC “Ashinsky Metal Plant”, Asha, Chelyabinsk region, Russia) perfectly. As a result, according to [31], the mutual diffusion of alloy components can be observed in the contact zone and nano- and micro-regions with body-centered cubic (BCC), and close-packed hexagonal (HCP) lattices can be formed. This provides good adhesion of the composite layers.

The Sn63Pb37 alloy was used for 5BDSR nanocrystalline tape (PJSC “Ashinsky Metal Plant”, Asha, Chelyabinsk region, Russia) fusing. To ensure adhesion, an indelible solder paste SD-318 (melting point 456 K) was used, consisting of RMA flux (rosin mild activated—slightly activated rosin) and solder powder Sn63Pb37, with particles of a spherical shape.

The sandwich “tape + fusible alloy + tape” was compressed in a specially designed mold and placed in a preheated one with the temperatures T = (423 ± 5) °C lower than the recrystallization/crystallization temperature of nanocrystalline/amorphous alloy, but sufficient to reduce the viscosity of the molten fusible alloy. The sample was isothermically annealed in the air environment for 5 min, and then slowly cooled down to room temperature. In the preparation of samples, it was necessary to ensure that the fusible component evenly covered the surface of the nanocrystalline/amorphous tape, and its thickness did not exceed 200 microns. The total thickness of the samples was 180 ± 9 microns. Samples with a transverse thickness difference of more than 10 microns were rejected.

**Figure 1:**
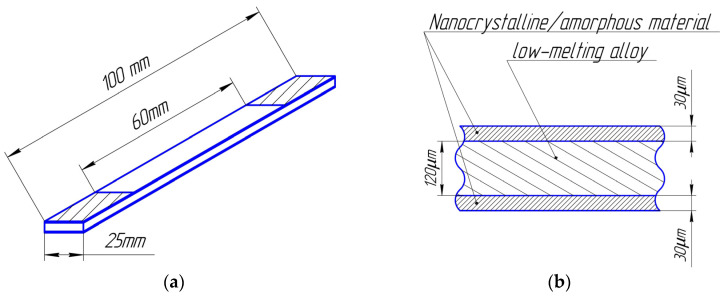
Composite sample model: (**a**) general view of the sample, the shaded areas of the sample were fixed in the clamps of the Instron 3365 universal testing machine; (**b**) a cross section of a composite sample consisting of two nanocrystalline/amorphous tapes connected by a low-melting metal alloy.

For mechanical testing, the laminated composite samples were fixed in the clamps of the Instron 3365 (supplied by Illinois Tool Works Inc., Glenview, IL, USA; equipment details available on the equipment website https://imc-systems.ru/about/partners/illinois-tool-works-inc/, accessed on 10 February 2022) universal testing machine in accordance with international testing standards [32,33]. The loading was carried out at a fixed deformation rate of 10–4 m/s. Figure 1a shows schematically the prepared sample in the clamps of the testing machine. Sample is based on a nanocrystalline alloy and a low-melting alloy, the arrangement of the layers is shown too (Figure 1b).

The prepared samples were tested for rupture during uniaxial stretching at temperatures of 77 K, 195 K and 293 K. The test samples were placed in solid CO_2_ (195 K) and liquid nitrogen (77 K) to maintain the set temperatures. Mechanical tests began one minute after the predetermined temperature was set, and the process was controlled using a low-temperature thermocouple. The control breaks of the tapes of nanocrystalline alloy 5BDSR and amorphous alloy 82K3XSR were carried out under the same conditions in order to make a comparative analysis and identification of the features of deformation and fracture of layered composites. To study the specific character of deformation and destruction in the diagrams obtained in the experiment, microscopy of samples after rupture was performed. In this research, we first studied the morphology of deformation and destruction in the area of branching cracks, in the area at the tops of stopped cracks and along the banks of cracks. An Axio Scope A1 microscope (supplied by LLC Carl Zeiss, German, Jena; equipment details available on the equipment website https://zeiss-solutions.ru/en/equipment/mikroskopiya/light-microscopy/zeiss-axio-scope-a1-light-microscope-for-biology/, accessed on 12 December 2023) was used to perform metallographic analysis of the samples surfaces.

## 3. Results

### 3.1. Experimental Results

The conducted mechanical tests revealed the features of deformation and destruction of a layered metal composite based on nanocrystalline/amorphous tapes under the conditions of cryogenic embrittlement.

**Figure 2:**
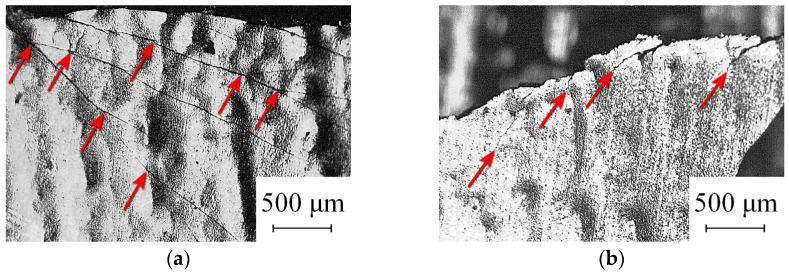
Micrographs in the fracture zone illustrating: (**a**) specifics of cracks branching, the arrows indicate the branching points; (**b**) specifics of destruction in the area of the vertices of branched and stopped cracks, the arrows indicate the vertices of stopped cracks.

Under tension, some samples of composite compounds were found to have a viscous nature of destruction, regardless of the test temperature. In contrast to comparison samples—individual nanocrystalline/amorphous samples that break into many parts (similar to brittle glass breaking)—the composite sample was mainly broken into two parts. There was a large number of branched and stopped cracks with areas of plastic deformation (Figure 2). Such crack growth indicates viscous fracture, in which some of the energy is used in plastic deformation, which increases the energy intensity of the fracture. Under the conditions of destruction caused by uniaxial stretching at 77 K, traces of plastic deformation of the amorphous tape as part of the composite compound were noted. In the conditions of similar tests involving one amorphous tape, the destruction was always brittle.

In the sample cooled to temperatures corresponding to cryogenic embrittlement, both regions with a morphology characteristic of brittle fracture and regions with quasi-brittle/viscous fracture of a layered composite are observed. In areas of brittle fracture, chips of amorphous material along planes perpendicular to the axis of tension were seen without external traces of macroscopic flow. Viscous fracture developed in the planes with maximum tangential stresses. Two zones with different crack morphologies were identified on the fracture surface—almost smooth cleavage areas and areas with a system of “intertwining” traces of localized plastic flow.

Tapes of nanocrystalline/amorphous alloys, unlike composite samples, become brittle when cooled from a temperature of 293 K to temperatures of 195 K and 77 K. The nature of their destruction changes significantly. Brittle fracture was observed in areas of rectilinear crack propagation. No stopped cracks were observed. The samples broke down into many small pieces.

**Figure 3:**
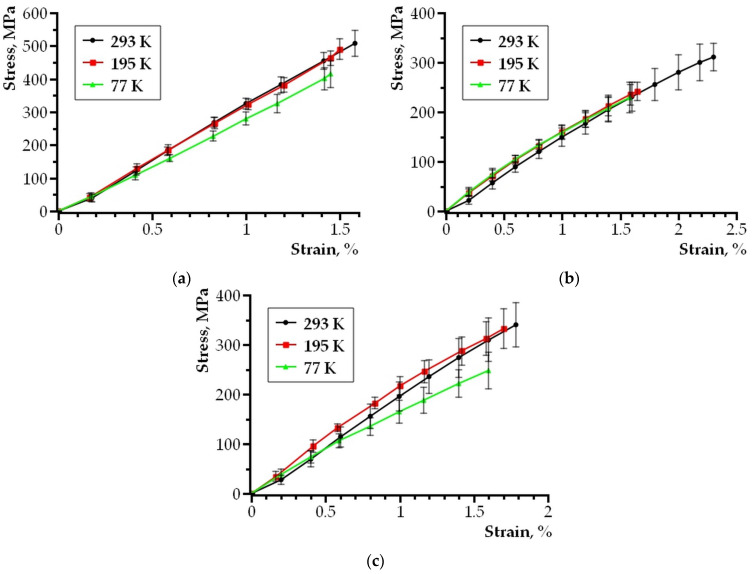
Stretching diagram of samples: (**a**) composite based on Sn63Pb37 alloy and 5BDSR nanocrystalline tape; (**b**) composite based on POSK 50-18 alloy and 82K3XSR amorphous tape; (**c**) composite based on Wood alloy and 82K3XSR amorphous tape.

Figure 3 shows the stretching diagrams of composite samples prepared in accordance with the procedure (described above) at temperatures of 293 K, 195 K, and 77 K. The analysis of the obtained dependence curves showed that cooling down to cryogenic temperatures leads to some deterioration in the mechanical properties of composite samples. However, the embrittlement effect of the prepared samples of layered composites turned out to be smaller than expected.

Figure 3a shows a stretching diagram of a composite based on the Sn63Pb37 alloy and a 5BDSR nanocrystalline tape. The tensile strength of composites with the Sn63Pb37 alloy at 293 K is 507 MPa on average, and the average elongation is 1.57%. The tensile strength of the material, when the temperature drops to 195 K, is 490 MPa on average, and the elongation is 1.50%. When the temperature drops to 77 K, the average tensile strength decreases to 415 MPa, and the elongation reduces to 1.45%.

Figure 3b shows the stretching graph of the composite for the POSK 50-18 alloy and the 82K3XSR amorphous tape at temperatures of 293 K, 195 K and 77 K. The tensile strength of composites based on the POSK 50-18 alloy and the amorphous 82K3XSR alloy at 293 K is 310 MPa on average, and the average elongation is 2.29%. The tensile strength of the material, when the temperature drops to 195 K, is 241 MPa on average, and the elongation is 1.65%. When the temperature drops to 77 K, the average tensile strength decreases to 229 MPa, and the elongation reduces to 1.58%.

Figure 3c shows the tensile diagram of an alloy-based composite. The ultimate strength of the material, when the temperature drops to 195 K, averages 241 MPa and the elongation is 1.65%. The tensile strength of composites based on Wood alloy and amorphous alloy 82K3XSR at 293 K is 340 MPa on average, and the average elongation is 1.78%. When the temperature drops to 195 K, the tensile strength increases to 332 MPa, and the elongation is 1.70%. When the temperature drops to 77 K, the average tensile strength decreases to 248 MPa, and the elongation remains at 1.60%.

### 3.2. Theoretical Analysis

The graphic dependence curves in Figure 3 show that the composite compound of nanocrystalline/amorphous film–fusible alloy retains the plastic character of destruction when the test temperature decreases to 77 K. This may be related to the fact that the crack growth in the composite is accompanied by local heating, whereby the area at the top of the crack heats up as a result of plastic deformation. This effect has been previously considered theoretically in [16] and is known as the “free volume” model. The model was proposed by Spapen; the plastic deformation of amorphous metal alloys is based on the concept of free volume. There is no regular crystal lattice in amorphous materials, so the atoms are randomly distributed. Within the framework of the “free volume” model, it is assumed that plastic deformation occurs due to a series of atomic jumps to the location of a single free volume [16]:

(1) $$\sigma =\frac{U-kT\ln M/\dot{\varepsilon }}{V},$$

where σ is the yield strength, $\dot{\varepsilon }$ is the deformation rate, U is the height of the potential barrier for the movement of dislocations, k is the Boltzmann constant, V is the activation volume, and M≈const.

The process of destruction is heterogeneous in nature. There are two stages of the process that determine the rate and mechanism of crack development. At the slow stage, fluctuations in the thermal motion of atoms stimulated by excessive local stresses at the crack tip are observed. Interatomic bonds are loosened, and a “free” fluctuation volume arises, which is a drain of released elastic energy. A heat source appears in this zone due to atomic jumps, so the material at the crack tip heats up and the mechanical properties of the sample change. There is a viscous course of the crack development process.

However, this stage is nonstationary. The appearance of thermal fluctuations with energies sufficient to break interatomic bonds leads to an increase in the crack growth rate. The crack converges into stationary mode. The destruction develops rapidly and becomes non-thermal and brittle. We emphasize that the change in the destruction mechanism is accidental.

In conducting uniaxial tensile tests, the composite compound retains the possibility of plastic deformation, even at 77 K. Such abnormal behavior of the material may be related to the peculiarities of plastic deformation occurring in nanoblasts at the boundary of a nanocrystalline/amorphous material and a low-melting crystalline metal alloy. It is assumed that according to the mechanism theoretically described in [34], there is a series of atomic jumps, leading, in turn, to local heating. Depending on the initial conditions of crack growth, fracture can be implemented by various mechanisms. If crack growth is slow initially, there will be a heated area in front of the crack tip. Thus, we can explain the presence of both viscous and brittle crack growth variants during the destruction of composite samples at 77 K.

The plastic nature of the fracture is apparently caused by the following factors. For the nanocrystalline/amorphous tapes and low-melting crystalline metal alloys used, the boiling point of liquid nitrogen turns out to be lower than the Debye temperature Θ, which can be estimated for these materials, taking into account the concentration of chemical components [35], where for each component, the temperature $\Theta_{i}$ is calculated, for example, according to the Lindemann Equation (2) [36]:

(2) $$\Theta_{i}=137\sqrt{\frac{T_{пл}^{i}}{A_{i}{V_{i}}^{2/3}}},$$

where $\Theta_{i}$ is the Debye temperature, $T_{пл}^{i}$ is the melting point, $A_{i}$ is the atomic mass, and $V_{i}$ is the atomic volume of the component.

Thus, in the initial stage of atomic relaxation, the heating of the composite near the crack tip is due to the absorption of phonon energy by the electrons of the material. As the local temperature of the atoms at the crack tip becomes higher, a further temperature increase can be considered within the framework of the theory of inhomogeneous plasticity of a layered composite, taking into account thermal fluctuations at the crack tip [37]. According to the Landau–Khalatnikov theory, the relaxation rate is defined as [38]:

(3) $$a_{n}\left(T,\sigma \right)=\omega_{0}\left[e^{-\frac{\left(E_{\eta }-\sigma V_{\eta }\right)}{kT}}+\Theta (\sigma -\sigma_{1})e^{-\frac{a}{a_{dB}}}\right],$$

where $a_{n}\left(T,\sigma \right)$ is the relaxation rate, which contains two terms of different physical nature. $E_{\eta }$ is the activation energy of atomic restructuring, σ is the mechanical stress, and $V_{\eta }$ is the activation volume of the cluster that has experienced atomic restructuring. Here, $a_{dB}(\sigma )$ is the de Broglie wavelength, a is the width of the potential barrier in the two-hole potential, $\omega_{0}\approx 10^{13}$ Hz, $\Theta (\sigma -\sigma_{1})$ is a theta function that is nonzero when the magnitude of the stress σ causes inelastic deformation, and $\sigma_{1}$ is the limit of proportionality.

The crack opening gradually decreases down to the interatomic distances near its tip. The smooth closing of the crack’s “shores” and the finiteness of stresses determine the “autonomy” of the crack tip. The diameter of the fluctuation volume and the length of the crack tip are almost the same [37]. Consequently, micro-damages may be accumulated in the micro-volume near the tip, leading to the occurrence of a loosening area with an additional free volume ahead of the crack front. Defects of this type affect the low-temperature properties of nanocrystalline/amorphous materials and can be considered as carriers of plastic deformation. The most well–known theory taking account of the presence of an inelastic zone in front of the crack is the Leonov–Panasyuk–Dugdale theory [39]. Under the conditions of low-temperature embrittlement of the composite, it can be assumed that a zone of forced plasticity occurs in front of the crack front, and this zone develops by overcoming the internal friction with the release of additional heat, which leads to local heating of the material in the vicinity of the crack tip. At each point of the volume in the vicinity of the crack tip, a heat source operates, and its power can be defined as [40]:

(4) Q˙=2μ¯τ2e−tτ·σ2·η(T−Θ),

where $\dot{Q}$ is the amount of heat released, $\bar{\mu }$ is the molar mass of the sample, τ is the delay time of forced plastic deformation, σ is the average plastic deformation at the crack tip, and η(T−Θ) is the Heaviside function.

On the other hand, as the temperature decreases, the heat capacity decreases, so the energy released in the nanoscale region at the crack tip is enough to warm it up to temperatures at which low-melting alloys remain ductile.

There is currently no uniform view on the mechanism of heterogeneous plasticity of nanocrystalline/amorphous materials and layered composites based on them [16]. The interface of the layered structures is known to have a significant influence on the mechanical properties of nanomaterials due to its complicated microstructure. Local heating may influence the microstructural defects and affect the embrittlement behavior of the layered structure. The presence of microdefects at the interface also contribute to plastic deformation features. According to [10], some relaxation of stresses was due to plastic deformation of the layered structure; we also observed that the embrittlement effect in the samples of layered composites was smaller than expected. Analysis of the deformation process on the scale of fluctuation volume in the growing cracks tips is required. Computer modeling of this process at both the continuum and atomic levels will reveal its features and determine the main mechanisms of forced plasticity of the investigated materials in a wide range of temperatures below room temperature. In this case, one of the issues is the analysis of the temperature distribution in the cracked sample.

### 3.3. Simulation Results

Taking into account the above-discussed theoretical concepts based on the analysis of physical phenomena [16,36,37,38,39,40] describing the fracture process in a nanocrystalline/amorphous material, a physical model of the heating of the region in the vicinity of the crack tip was formulated. The heat transfer by the mechanism of thermal conductivity with internal heat sources in the investigated solid material is described by the differential equation of thermal conductivity (Fourier differential equation). Free convection occurs on the outer surface of the sample (except for the crack surface), which is described by the Newton–Richman Equation (5) [41]:

(5) $$q=\alpha (T_{c}-T_{f}),$$

where q [W/m^2^] is the heat flux density, $T_{c}$ [K] is the surface temperature of a solid, $T_{f}$ [K] is the ambient temperature, and α [W/(m^2^ K)] is the heat transfer coefficient by convection.

As far as the problem of thermal conductivity is concerned, there are four types of boundary conditions and their combinations. This is enough to describe any interaction of a virtual sample with the environment [40]. Since according to the results of preliminary model experiments the excess temperature does not have time to spread to the surface of the sample, it is true that $T_{c}=T_{f}$, and then q=0. Then, the boundary conditions of the third kind (Newton’s conditions) degenerate into boundary conditions of the second kind (Neumann’s conditions), and are used in further modeling [41,42,43]. The validity of the application of these boundary conditions is also confirmed by the fact that no heating of these surfaces was detected during the experiment. These boundary conditions define the distribution of the heat flux in the *G* region and its change over time: $-\lambda {\left.\frac{\partial T}{\partial n}\right|}_{G}=q\left(G,\tau \right)$, where *G* is a set of points on the surface of the body to which boundary conditions of the second kind are applied, *τ* is the time starting from the beginning of modeling, *T* is the temperature field inside the body, n is the normal vector to the surface of *G* directed from the body, and *λ* is the thermal conductivity coefficient of the body material.

**Figure 4:**
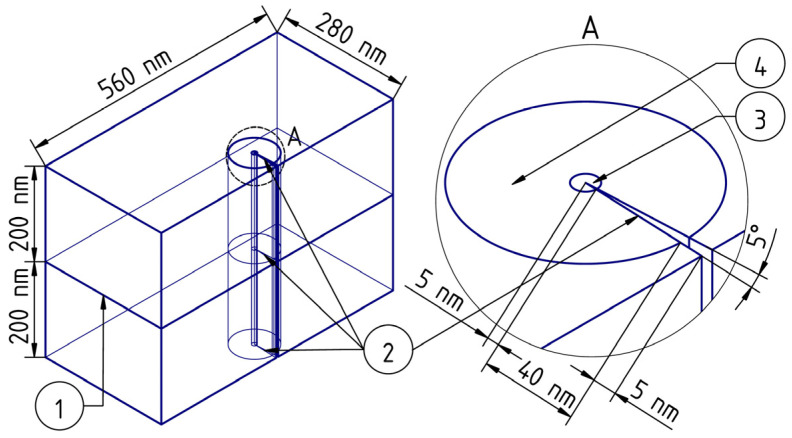
Geometry of the simulated sample section: 1—the interface between an amorphous nanocrystalline ribbon and a low-melting metal alloy, 2—a crack, 3—a heat release region, 4—an area with an increased density of the finite element method grid. The remote element *A* shows the area at the top of the crack.

While modeling the fracture of the sample, it is necessary to consider the thermal effects at the tip of the growing crack. The correctness of the specified conditions consists of accounting for the rate and nature of the crack growth (with viscous fracture, the curvilinear nature of crack growth and multiple branching are observed), in view of the thermodynamic average velocity of the gas-phase molecules (the average velocity of nitrogen molecules will be ≈240 m/s). In our case, the molecules of the gas phase are nitrogen molecules at a temperature above the boiling point by 3–5 degrees (80–82 K). At the same time, in model experiments, the average crack growth rate was set in the range of 5–500 m/s (much less than the speed of sound in this medium). Taking into account the kinematic/dynamic viscosity of nitrogen and the curved trajectory, we find that no convection cooling is observed at the crack tip during a time of the order of $10^{-10}–10^{-12}$ s on the crack surface. Thus, it is possible to proceed to the consideration of the thermal conductivity equation in view of meeting the criteria of the physical model discussed above [40,41,42,43]. Heat transfer by the mechanism of thermal conductivity in a solid material with internal heat sources is described by the differential equation of thermal conductivity (6):

(6) $$c\rho \frac{\partial T}{\partial t}=\left\{\begin{array}{c} Q+\nabla (\lambda \nabla T),\;\;(x,y,z)\in G\\ \nabla \left(\lambda \nabla T\right),\;\;(x,y,z)\notin G \end{array}\right.,$$

where G is the selected area at the crack tip (see Figure 4), T is the temperature (K), λ is the coefficient of specific thermal conductivity (W/(m^2^·K)), c is the specific heat capacity (J/(kg·K)), ρ is the density (kg/m^3^), and Q is the thermal power of internal heat sources (J/(s·m^3^)).

It is possible to solve the differential equation of thermal conductivity for a body with a complex geometric shape using only numerical methods. In this investigation, (using the finite element method), the original complex structure was (broken down) split into simple elements using the finite element method. Then, a system of algebraic equations was compiled based on the grid of the finite element method and the equation of thermal conductivity [40,41,42,43]. This system of equations was solved using a standard algorithm. A computer simulation of the experiment was carried out using software packages. FreeCAD Version 0.20 (https://www.freecad.org/, accessed on 20 February 2023) is responsible for creating a 3D geometry model, interacting with other software packages within the framework of this investigation, processing the obtained results and visualization. Gmsh Version 4.10.3 (https://www.freecad.org/?lang=ru, accessed on 20 February 2023) generates a finite element method grid based on a 3D model. ElmerGUI Version 9.0 (https://www.csc.fi/web/elmer, accessed on 20 February 2023) sets the parameters and settings of the simulation and solves the task. The simulation was carried out using the finite element method on the basis of the thermal conductivity equation for solids. The 3D model was created using FreeCAD, a finite element method grid was created using Gmsh (defining areas with increased density of the finite element method grid), the grid was imported in the ElmerGUI program, modeling parameters were set and modeling was performed, and the results were exported to the FreeCAD program for final processing and visualization.

A schematic representation of the simulated sample is shown in Figure 4. The area of the crack tip local self-heating caused by plastic effects is represented on the remote element *A* with a large magnification (element 3 of Figure 4). The self-heating area and the material around it are characterized by the highest temperature gradient; therefore, the density of the finite element method grid is increased there (element 4 of Figure 4). Geometrically, this area represents a cylinder, one of the bases of which is shown in the remote element *A* of Figure 4. According to the preliminary model experiments, it was found that the maximum temperature gradient was fixed in the space bounded by the cylinder with a base diameter of 90 nm, as shown in Figure 4. In Figure 4, the nanocrystalline/amorphous ribbon is on top, while the fusible metal alloy is on the bottom.

A small sample volume (280 × 560 × 400 nm) of a layered composite based on a nanocrystalline/amorphous ribbon and a low-melting crystalline alloy in the area of a symmetrical edge crack with a depth of 50 nm was simulated. The region in which the free fluctuation volume occurs is localized around the crack tip; therefore, it is assumed that the effect of residual stresses on the EDGE of the sample becomes negligible as a given depth is reached. The crack opening angle was set in the range of 5–15°. According to the physical representations discussed above, the diameter of the atomic jump region was assumed to be 10 nm.

**Figure 5:**
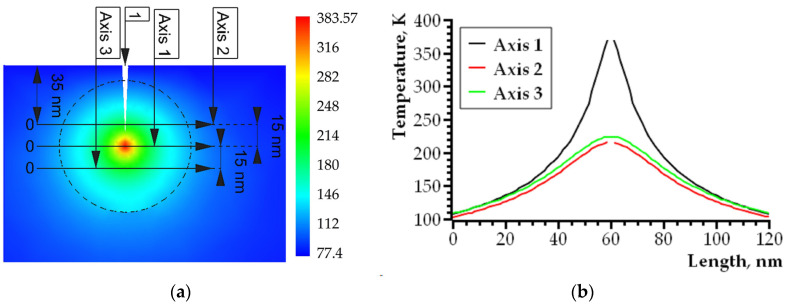
The features of the temperature distribution in the area of the crack tip: (**a**) scalar temperature field and temperature scale, 1—crack; (**b**) dependence curves of the temperature distribution along axes No. 1, 2, 3.

A scalar field of temperature distribution in a sample with a crack was obtained as a result of the simulation. Figure 5 shows the specifics of the temperature field distribution in the plane perpendicular to the plane of the growing crack. The temperature distribution was analyzed not in the entire sample but only in its central part in the vicinity of the crack tip. Figure 5a shows a central fragment with a temperature gradient. Axes No. 1, 2, 3 (Figure 5a) are built on the lines formed by the cross-section of the sample, with the planes perpendicular to plane 1 shown in Figure 4. Axis 1 passes through the tip of the crack. In a small area near the crack tip, high mechanical stresses and large temperature gradients are observed; thus, the correct calculation by the finite element method using Equation (6) is impossible. Thus, a correct calculation using the finite element method, using Equation (6), is impossible in this very small area due to the limited applicability of the classical heat transfer equations. Graph 5b shows a gap in dependencies No. 1 and 2. This gap corresponds to the area between the surfaces of the crack. Therefore, a gap has been made in the area of the crack tip for dependence curve No. 1 (Figure 5b). Temperature changes were recorded along axes No. 1, 2, 3 (from left to right). The characteristic features of the temperature change along these axes are illustrated by the dependence curves shown in Figure 5b.

The simulation results shown in Figure 5 demonstrate the local character of the heating at the crack tip. This confirms the validity of choosing a high-density grid of the finite element method only in the crack tip region. The temperature drops rapidly with the distance from the crack tip. Therefore, in the case of high crack growth rates, a transition from the viscous fracture mode to the brittle one is possible. This is consistent qualitatively with the results of the experiment, since under the same experimental conditions, morphology was recorded corresponding to both the viscous nature of the fracture (Figure 2) and the brittle one. Higher temperature values along axis No. 3 and lower values along axis No. 1 indicate the influence of the sample surface cooled to 77 K. Thus, for a slowly growing crack, an effective temperature can be introduced (in this model it is about 300 K). This temperature determines the local mechanical properties of the material and ensures the viscous nature of the fracture.

**Figure 6:**
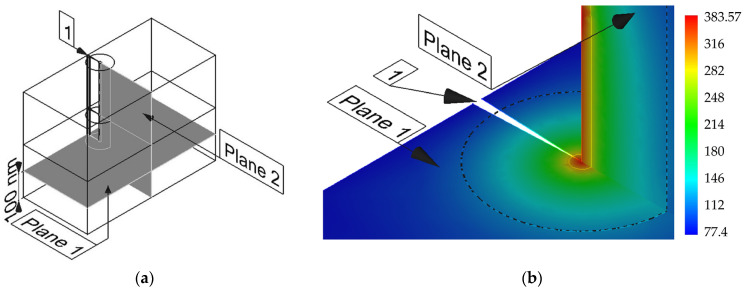
(**a**) The geometry of the sample in the area of the crack tip, indicating the areas where the temperature distribution was analyzed; (**b**) scalar temperature fields in planes 1 and 2; 1—crack.

**Figure 7:**
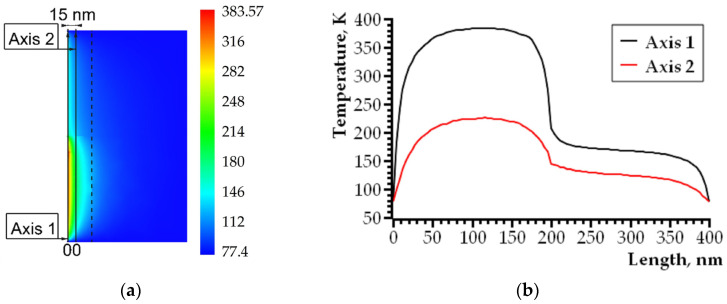
The heating pattern of the material in front of the growing crack tip: (**a**) plane No. 2 of Figure 6a, the dotted line bounds the area with the increased density of the finite element method grid; (**b**) the quantitative temperature change along axes No. 1 and 2 is shown.

In addition to the features of heating in the plane perpendicular to the plane of the crack (Figure 5 and Figure 6a), it is necessary to analyze the temperature distribution along lines parallel to the plane of the growing crack and located in front of its tip (Figure 6 and Figure 7). These lines intersect the melting plane of nanocrystalline/amorphous tapes and fusible material at right angles (Figure 4 and Figure 6a). Thus, it is possible to identify the features of heating the material in the contact area of an amorphous nanocrystalline and low-melting alloy. It seems important to understand the process of plastic deformation initiation both on the basis of Equation (1) and in view of the thermal activation nature of elementary acts of plastic deformation. Figure 6a shows the geometry of the analyzed area. The heating pattern in plane 2 is shown in Figure 6b and Figure 7a. The dependence curves No. 1 and No. 2 shown in Figure 7b are built on axes lying in plane 2 and perpendicular to plane 1 (Figure 6a). Herein, axis No. 1 is located at a distance of 1.5 nm from the crack tip, and axis No. 2 is located at a distance of 15 nm from it (shown in Figure 7a).

Figure 6a shows the heating patterns of the material near the crack tip in both plane 1 and plane 2. Plane 1 (Figure 6a), for which the heating pattern of the material is shown (Figure 5a and Figure 6b), is located below the plane of fusion of a nanocrystalline/amorphous material and a fusible alloy (Figure 4). Figure 6b and Figure 7 visualize the heating pattern of the material when crossing the boundary region. According to computer simulation data, the heating features change significantly during the transition from a low-melting alloy to a nanocrystalline/amorphous material.

When planning the experiment, different materials were selected. Amorphous and nanocrystalline tapes were used to form the outer layers, and various low-melting alloys were used to make the core of the layered composite. This was in order to experimentally identify a combination of materials and structures in the boundary layer of the composite that provides the best mechanical properties when the temperature decreases from 293 K to 77 K (Figure 3). For all composites, there is a decrease in the ultimate mechanical stress of fracture and in the relative elongation of the sample before fracture. However, the decrease in these mechanical characteristics is more significant for composites based on amorphous tapes. σ_st_ is reduced by about 26% for a composite with a POSC of 50–18 and σ_st_ is reduced by 27% for a composite with a Wood alloy. While for a composite based on nanocrystalline alloy 5BDSR, σ_st_ is reduced by only 18%. The relative elongation of the sample before destruction for composites based on amorphous tape with POSK 50-18 is 31%; for those with the Wood alloy, the relative elongation is 11%; and for composites made of nanocrystalline tape, it is 8%. Accordingly, the best conditions for the initiation of self-heating occur at the boundary of a low-melting alloy and an amorphous nanocrystalline 5BDSR tape. In fact, any multicomponent nanocrystalline alloy contains disordered regions between the crystallites, being an amorphous nanocrystalline [1,26]. During uniaxial stretching, its deformation is inhomogeneous, and is accompanied by additional rotational effects at the nanocrystal boundary. Taking into account the above theoretical analysis (Formulas (1)–(4)), this facilitates the process of self-heating initiating at the top of a slowly growing crack. This leads to better mechanical properties at 77 K for a layered composite based on nanocrystalline tapes.

The initiation of self-heating at the boundary of a nanocrystalline/amorphous and low-melting alloy has a significant effect on the process of deformation and destruction. The energy released in front of the crack tip is sufficient to heat the material by 100–200 K, with a transition to viscous fracture. This process is essentially probabilistic, as it is possibly determined by the nature of crack growth at the beginning of the fracture process. If the crack growth is preceded by atomic rearrangements, the fracture will be viscous even at the cryogenic temperature of the composite sample as a whole. Thus, the creation of composite “nanocrystalline/amorphous film-fusible metal alloy-nanocrystalline/amorphous film” that are able to maintain the viscous nature of destruction requires the identification of the physical patterns of self-heating initiation in nanoblasts at the boundary of nanocrystalline/amorphous and crystalline phases.

## 4. Conclusions

1. The effect of self-heating at the growing crack tip is realized at the nanoscale boundary layer between nanocrystalline/amorphous metal alloys and crystalline fusible alloys. This effect was detected in a temperature range of 77–293 K. The local heating zone at the crack tip ensures the viscous nature of the composite fracture by the main crack and the inhibition of fracture for branched cracks. Self-heating of the material at the moment of crack growth initiation is localized at the boundary layer (between the nanocrystalline/amorphous and crystalline phases), and then spreads to the entire area at the top of the growing crack. The effect of self-heating at the crack tip can change the nature of fracture from brittle to viscous even at cryogenic temperatures.
2. A physical model of deformation and fracture at the boundary of nanocrystalline/amorphous and crystalline phases in thin-layer composite compounds is proposed. The simulation results confirm the exothermic crack growth in the entire temperature range of 77 K, 195 K and 293 K under uniaxial stretching of the sample. The model suggests the origin of a crack at the interface between two components of a nanocrystalline/amorphous film and a low-melting metal alloy with further propagation deep into the layered composite. As a result of a sharp decrease in the heat capacity of the material layers at cryogenic temperatures, atomic jumps at the tip of the crack opening, leading to self-heating, cause local heating to temperatures corresponding to plastic deformation of the material in the region of high mechanical stresses. The physical model of the process is described using classical equations of thermal conductivity and computer simulation performed by the standard finite element method. In addition, the main conclusions of the proposed physical model are in good agreement with the experimental data obtained on similar layered structures.
3. The boundary layer between the amorphous nanocrystalline and microcrystalline metallic phases can have high plasticity at cryogenic temperatures and provide fracture toughness when critical stresses are exceeded with the appearance of microcracks. Consequently, such composite layered structures can maintain a high strength at cryogenic temperatures and have the ability to prevent brittle/catastrophic destruction by increasing the energy intensity of destruction.

## Data Availability

Data are contained within the article.
